# Electroencephalography as a Biomarker for Functional Recovery in Spinal Cord Injury Patients

**DOI:** 10.3389/fnhum.2021.548558

**Published:** 2021-04-09

**Authors:** Marcel Simis, Deniz Doruk Camsari, Marta Imamura, Thais Raquel Martins Filippo, Daniel Rubio De Souza, Linamara Rizzo Battistella, Felipe Fregni

**Affiliations:** ^1^Hospital das Clinicas HCFMUSP, Faculdade de Medicina, Universidade de São Paulo, São Paulo, Brazil; ^2^Department of Psychiatry and Psychology, Mayo Clinic, Rochester, MN, United States; ^3^Neuromodulation Center, Spaulding Rehabilitation Hospital, Harvard Medical School, Boston, MA, United States; ^4^Universidade Paulista and Universidade Nove de Julho, São Paulo, Brazil

**Keywords:** spinal cord injury, electroencephalography, rehabilitation, biomarkers, neuroplasticity

## Abstract

**Background:**

Functional changes after spinal cord injury (SCI) are related to changes in cortical plasticity. These changes can be measured with electroencephalography (EEG) and has potential to be used as a clinical biomarker.

**Method:**

In this longitudinal study participants underwent a total of 30 sessions of robotic-assisted gait training (RAGT) over a course of 6 weeks. The duration of each session was 30 min. Resting state EEG was recorded before and after 30-session rehabilitation therapy. To measure gait, we used the Walking Index for Spinal Cord Injury Scale, 10-Meter- Walking Test, Timed-Up-and-Go, and 6-Min-Walking Test. Balance was measured using Berg Balance Scale.

**Results:**

Fifteen participants with incomplete SCI who had AIS C or D injuries based on American Spinal Cord Injury Association Impairment Scale classification were included in this study. Mean age was 35.7 years (range 17–51) and the mean time since injury was 17.08 (range 4–37) months. All participants showed clinical improvement with the rehabilitation program. EEG data revealed that high beta EEG activity in the central area had a negative correlation with gait (*p* = 0.049; β coefficient: −0.351; and adj-*R*^2^: 0.23) and balance (*p* = 0.043; β coefficient: −0.158; and adj-*R*^2^:0.24) measured at baseline, in a way that greater high beta EEG power was related to worse clinical function at baseline. Moreover, improvement in gait and balance had negative correlations with the change in alpha/theta ratio in the parietal area (Gait: *p* = 0.049; β coefficient: −0.351; adj-*R*^2^: 0.23; Balance: *p* = 0.043; β coefficient: −0.158; and adj-*R*^2^: 0.24).

**Conclusion:**

In SCI, functional impairment and subsequent improvement following rehabilitation therapy with RAGT correlated with the change in cortical activity measured by EEG. Our results suggest that EEG alpha/theta ratio may be a potential surrogate marker of functional improvement during rehabilitation. Future studies are necessary to improve and validate these findings as a neurophysiological biomarker for SCI rehabilitation.

## Introduction

Spinal cord injury (SCI) is one of the most important causes of permanent disability with a major social and economic impact on the affected population. Patients with SCI have a higher mortality risk, which can be 28.8 times greater than the reference population (Leite et al., 2019). The worldwide prevalence of SCI is estimated to be 440 to 526 per million people, with an incidence rate varying from 13.0 per million to 163.4 per million people and reaching 220.0 per million people in under-developed countries (Kang et al., 2018).

Rehabilitative therapies in SCI have significantly improved over the past decades with the development of better rehabilitation protocols and new technologies such as robotic-assisted gait training (RAGT). Despite these advances, there are still a significant number of patients with severe impairment who fail to show any significant improvement with these interventions (Scivoletto et al., 2011; Nam et al., 2017). Understanding the mechanisms of functional recovery in SCI and developing surrogate markers of treatment response are crucial for the development of innovative approaches and tailored treatments for SCI patients. New evidence suggests that neuroplasticity has an essential role in restoring sensory, motor and autonomic functions, by the reorganization of circuits at the level of the SCI, and in the cortical areas of the brain (Liu et al., 2012). Both adaptive and maladaptive neuroplasticity in SCI have been previously investigated using different neuroimaging techniques and quantitative electroencephalography (qEEG).

Studies with functional Magnetic Resonance Imaging suggest that SCI patients have alterations of functional connectivity in different areas of the encephalon, including the primary motor cortex (M1), the premotor cortex (PMC), the supplementary motor area (SMA), the thalamus (TH), the cerebellum, and the right orbitofrontal cortex (OFC). These changes are thought to be related to functional and structural reorganization following the injury to efferent and afferent neural pathways (Athanasiou et al., 2017) and have been shown continue to evolve over time following the injury (Hawasli et al., 2018). Moreover, it is suggested that SCI patients with poorer recovery have decreased functional connectivity between the primary motor cortex and other higher order motor areas such as SMA and PMC as compared to the patients with good recovery (Hou et al., 2016).

Another instrument to study cortical networks is qEEG due to its accessibility, relatively low-cost application and high temporal resolution as compared to other neuroimaging techniques. QEEG measures have been investigated in numerous neuropsychiatric conditions to guide or predict treatment response. Another two important areas where qEEG have provided important insights into are human learning (Etnier et al., 1996; Smith et al., 1999; Kiefer et al., 2014) and cognition (Trammell et al., 2017).

Studies in SCI have so far shown decreased EEG alpha power, lower peak frequencies, and higher beta power in SCI subjects (Tran et al., 2004; Herbert et al., 2007; Doruk et al., 2017). Additional findings in SCI included abnormal or absence of somatosensory- evoked potentials (Lewko et al., 1995; Cheliout-Heraut et al., 1998; Spiess et al., 2008; Kuhn et al., 2012), altered event- related synchronization-desynchronization (Muller-Putz et al., 2007; Gourab, 2010; Cremoux et al., 2013; Müller-Putz and Kaiser, 2014), and cortical network changes (Mattia et al., 2006; De Vico Fallani et al., 2007, 2008; Mattia et al., 2009). A longitudinal study evaluating the event-related desynchronization (ERD) over the motor cortex within the first months following the injury showed that alpha and beta ERD evolution after SCI is negatively correlated with the clinical progression of the patients (Lopez-Larraz et al., 2015).

Advances in signal analysis methods have made it possible to use qEEG to index changes in M1, somatosensory cortex and in the parietal cortex that might be related to the adaptive and maladaptive reorganization of the neural networks after SCI. In a recent EEG study, analysis of functional connectivity within the sensorimotor networks during multiple motor imagery tasks showed that SCI patients had decreased connectivity within the sensorimotor network. SCI patients also had increased local processing during the late stages of imagery tasks which was attributed to adaptive compensatory mechanisms and plastic changes following injury (Athanasiou et al., 2018). In another study, compared to healthy controls, subjects with tetraplegia showed enlargement of functional networks and differences in functional interactions within these networks while performing an attempted movement (Mattia et al., 2009).

Current evidence suggests that EEG can be a useful tool to evaluate functional impairment and neuroplastic changes related to SCI. Previous studies have shown that qEEG measures may also be useful in predicting motor recovery following robot assisted rehabilitation in other conditions such as stroke (Trujillo et al., 2017). However, there is a lack of longitudinal studies that correlate changes in EEG with functional improvement after rehabilitation therapy in SCI. Therefore, in this study we aimed to identify the longitudinal changes in brain activity measured by EEG that are related to the functional improvement in SCI patients who have received rehabilitation therapy.

We hypothesized that an injury to corticomotor and somatosensory pathways would lead to neuroplastic changes in the central and parietal areas of the cortex that can be indirectly measured by the changes in neural oscillations at the alpha and beta frequencies measured by EEG. Based on the previous studies, we mainly focused on the brain activity in the alpha and beta frequency bands as these would be the most relevant EEG measures related to motor function and functional recovery. Other EEG bandwidths and alpha/theta ratio were tested in an exploratory analysis (Tran et al., 2004; Herbert et al., 2007; Doruk et al., 2017).

## Materials and Methods

This study was approved by the Ethics Committee for Analysis of Research Projects of University of São Paulo Medical School.

### Sample Characteristics

This study included 15 participants with incomplete SCI admitted to the Institute of Physical Medicine and Rehabilitation (IMREA) of University of São Paulo. Written informed consent was obtained from each participant. The inclusion criteria were: (1) diagnosis of incomplete SCI (rated as AIS C or D in American Spinal Cord Injury Association Impairment (ASIA) scale), (2) age between 16 and 70, and (3) one to 36 months after the injury.

The exclusion criteria were: (1) having cardiac pacemaker, (2) unstable angina or other decompensated heart disease, (3) chronic obstructive pulmonary disease, (4) dysreflexia of autonomic system, (5) tracheostomy, (6) presenting deformity and stiffness of the hip joint, knee (20° or more of flexion) and ankle (10 or more plantar flexion), (7) restricted range of motion, (8) lack of cardiovascular conditioning or disabling fatigue, (9) body weight greater than 135 kg, (10) body height greater than 2 meters, (11) leg length difference greater than 2 cm, (12) osteoporosis, (13) instable fracture in lower extremity, and (14) presence of decubitus ulcer of lower extremity.

### Rehabilitation Program

Participants went through the IMREA rehabilitation program, for about 8 weeks, consisted of six weekly sessions of 60 min of physical therapy, occupational therapy, and physical fitness, as well as two weekly 60-min sessions of nursing, nutrition, psychology and social work. For locomotion therapy, the Lokomat system (Hocoma AG, Switzerland), which is a RAGT, was used. A trained therapist conducted all sessions.

The Lokomat therapy was performed five times a week over a course of 6 weeks (for a total of 30 sessions). Each session lasted for 30 min. The training load and speed were gradually increased based on tolerability. The supporting body starts with 50% of the patient body weight, and it decreased by 10% every 2 weeks. The Lokomat guiding force varied between 90–75%, based on the patient’s capacity to perform active movements. The progression of gait speed was adjusted during the training period based on the tolerance level of the participant. The treatment was combined with the conventional gait therapy without the use of the suspension device used in gait training.

### Demographic and Clinical Variables

Information on age, gender, time since the injury, level of the lesion, and medication use was obtained from all subjects. Participants were classified according to the ASIA to determine the ASIA Impairment Scale (AIS). Participants were categorized into AIS “C” or “D,” depending on their ability to move at least half of the key muscles below the single neurological level, with adequate strength or not.

To measure gait, we used the Walking Index for Spinal Cord Injury (WISCI II) Scale, 10-Meter- Walking Test (10MWT), Timed-Up-and-Go (TUG), and 6-Min-Walking Test (6MWT; Ditunno et al., 2000; van Hedel et al., 2005; Bohannon, 2006; Jackson et al., 2008). Individuals without walking ability were scored a speed value of 0 m/s. To measure balance, we used Berg Balance Scale (BBS; Berg et al., 1989). The evaluations were performed by the same rater at the baseline (i.e., prior to any Lokomat training), and after 30 sessions of training. Participants could use assistive devices and braces during the tests, but not harness systems, parallel bars or other support systems.

### Electroencephalography

Resting-state electroencephalography (EEG) was recorded for 20 min using a 128-channel EEG cap with active electrodes (Acti-Champs, PyCorder, Brainvision LLC^®^). The acquisition was performed before and after 30-session rehabilitation therapy. All recordings were obtained eyes closed. The EEG data were analyzed visually by an EEG expert clinical neurophysiologist to exclude the existence of epileptiform discharges.

For the offline analysis, we used a high-pass filter of 1 Hz and low-pass filter of 40 Hz, followed by manual artifact detection and rejection by a blinded assessor. Then, the data were exported and analyzed offline with EEGLab (Delorme and Makeig, 2004) and MATLAB (MATLAB R2012a, The MathWorks Inc. Natick, MA, United States, 2000).

The artifact-free data was processed using Fast Fourier transformation (averaged windows of 5 s with 50% overlap) to calculate power (μV^2^) for the following EEG bands: theta (4–8 Hz) and alpha (8–13 Hz) and the sub-bands: low alpha (8–10 Hz), high alpha (10–13 Hz), low beta (13–20 Hz), and high beta (21–30 Hz). Electrodes representing cortical areas were selected and averaged over for the following regions: frontal (F1, F2, F3, F4, F5, F6, AF3, AF4, FC3, and FC4), central (C1, C2, C3, C4, C5, and C6), parietal (P1, P2, P3, P4, P5, and P6), temporal (T1, T2, T3, T4, T5, T6, T7, T8, T9, and T10), and occipital (O1, O2, and Oz) areas.

### Statistical Analysis

#### Functional Outcomes

Due to our small sample size, a non-parametric test (Wilcoxon Signed Rank) was performed to compare pre- and post- treatment gait and balance measures (WISC, 10MWT, 6MWT, TUG, and BBS). Data were expressed as mean and standard deviation for the analysis. Participants who could not perform the tests were not included in the analyses. For the demographic and clinical characteristics gender, AIS classification (C or D) and level of injury (cervical or bellow cervical) were treated as binary variable; and time since lesion (in months) and age (in years) as continuous variables. We conducted univariate analysis to assess the relationship between functional outcomes and demographic/clinical characteristics.

#### Functional Outcomes and EEG

##### Functional outcomes as a predictor of baseline EEG

First, the normality of EEG data was tested using histograms. After confirming that the data have approximately normal distribution, we conducted separate univariate linear regression analyses where the independent variable was one of the functional outcomes (WISCI or BBS measured at baseline) and the dependent variable was the baseline EEG power [separately for each frequency bands (e.g., alpha, low beta), or the alpha/theta power ratio (alpha/theta)] to assess if there is any association between functional outcomes and EEG measures at baseline. Each dependent EEG variable tested one at a time. The initial analyses were performed only for the average of central electrodes (related to primary motor cortex) and parietal electrodes (related to primary somatosensory cortex), since our hypothesis was that the expected EEG changes would be secondary to the lesion in the corticomotor and somatosensory pathways, and therefore, these areas would be the most likely to show alterations in EEG activities related to functional outcomes.

To test for the hypothesis that baseline EEG measures can predict functional improvement, we performed analyses where the independent variable was the improvement of WISCI or BSS calculated as the difference between post-rehabilitation – pre-rehabilitation, (ΔWISCI or ΔBSS) and the dependent variable was the baseline EEG power.

##### Functional outcomes as a predictor of change in EEG

To test the hypothesis that the change in brain activity measured by EEG (ΔEEG) is related to functional improvement (ΔWISCI or ΔBSS), we tested univariate models where the change in EEG activity [calculated as the difference between post-rehabilitation – pre-rehabilitation, (ΔEEG)] in specific cortical areas and in each frequency band (e.g., central alpha power post-rehabilitation minus central alpha power pre-rehabilitation) was the dependent variable, and the improvement of WISCI or BBS (ΔWISCI or ΔBSS) was the independent variable.

To test the specificity of the cortical area for the significant EEG variables, we also tested other brain regions (frontal, temporal and occipital; besides central and parietal). These other cortical areas were only tested for the EEG variables that were significant in the initial analyses since the analyses were hypothesis-driven. For the statistical analyses, we used Stata Statistical Software 15 (StataCorp LLC), and the significant level was *p*-value < 0.05.

## Results

Fifteen participants with incomplete SCI who had had AIS C or D injuries based on ASIA classification were included in this study. Mean age was 35.7 years (range 17–51) and the mean time since injury was 17.08 (range 4–37) months. Lesion levels of were cervical (*n* = 11), lumbar (*n* = 1), and thoracic (*n* = 3). Participants were taking medications due to clinical comorbidities, including chronic pain, depression, urinary incontinence, and spasticity. One of the participants did not have an EEG recording at baseline and was excluded from the analysis.

### Functional Outcomes

#### Functional Outcomes

The analyses of functional change (pre vs. post-treatment) showed significant improvement of gait measured by the scales WISCI (*p* = 0.009), 6MWT (*p* = 0.029), and TUG (*p* = 0.037), but the improvement was not significant for 10MWT (*p* = 0.093). There was also a statistically significant improvement for balance measured by BBS (*p* = 0.007). Because of the absence of a control group, it was not possible to state that the improvement was related to intervention. Due to severe gait impairment, 5 of 14 could not perform the test 6MWT, 10MWT, and TUG, and were excluded from the analyses. Table 1 summarizes the results.

**TABLE 1:** Improvement in functional outcomes.

	Pre mean (±SD)	Post mean (±SD)	*p*-value
WISCI	9.4 (8.1)	11.3 (8.2)	*p* = 0.009*
6MWT (m)	137.9 (105.3)	194.4 (105.4)	*p* = 0.029*
10MWT (s)	40 (47.9)	30.6 (35.1)	*p* = 0.093
TUG (s)	52.1 (54.0)	40.6 (62.5)	*p* = 0.037*
BBS	24.9 (20.4)	29 (22.5)	*p* = 0.007*

*SD: standard deviation; Asterisks represent *p*-values smaller than 0.05.*

### Functional Outcomes as a Predictor of EEG Variables

#### Baseline EEGs

For the selected cortical areas (central and parietal), only high beta power in the central area had a normal distribution and was considered in this analysis. Baseline high beta EEG power in the central area (average of central electrodes) was predicted by the baseline measurements of WISCI (*p* = 0.010; β coefficient: −0.003; and adj-*R*^2^: 0.39) and BBS (*p* = 0.012; β coefficient: −0.001; and adj-*R*^2^: 0.31).

Although our hypothesis was related to central and parietal areas, we tested other cortical areas for the same EEG variables to test whether the results were specific to these brain regions or represents a more global change in brain activity. Results are summarized in Table 2.

**TABLE 2:** Baseline EEG predicted by functional outcomes.

Power bandwidth High beta	WISCI *p*-value	BBS *p*-value
Frontal	0.025*	0.067
Temporal	0.980	0.234
Central	0.010*	0.012*
Parietal	0.785	0.744
Occipital	0.286	0.435

*Asterisks represent *p*-values smaller than 0.05.*

#### Changes in EEGs [Post-Rehabilitation – Pre-Rehabilitation, (ΔEEG)]

In the central area, ΔEEG for all bands had a normal distribution. In the parietal area, only Δ alpha/theta had a normal distribution and was considered in this analysis. Gait improvement measured by WISCI (**Δ** WISCI) had a negative correlation with the change in alpha/theta ratio in the parietal area (*p* = 0.049; β coefficient: −0.351; and adj-*R*^2^: 0.23). The improvement in balance (Δ BBS) had a negative correlation with the change in alpha/theta ratio in the parietal area (*p* = 0.043; β coefficient: −0.158; and adj-*R*^2^:0.24). Results are summarized in Table 3.

**TABLE 3:** Changes in EEG predicted by improvement in functional outcomes.

Δ Power bandwidth	Δ WISCI		Δ BBS	
	Central	Parietal	Central	Parietal
Δ Theta	0.960	NT	0.900	NT
Δ Alpha	0.082	NT	0.053	NT
Δ Low alpha	0.147	NT	0.061	NT
Δ High alpha	0.052	NT	0.131	NT
Δ Low beta	0.263	NT	0.941	NT
Δ High beta	0.694	NT	0.851	NT
Δ Alpha/Theta	0.071	0.049*	0.095	0.043*

*NT: Not tested (since it was not normally distributed); Asterisks represent *p*- values smaller than 0.05.*

The significant results in the parietal area (mentioned above) were also tested for frontal, temporal and occipital areas. None of the other cortical areas was found significant, suggesting the high specificity of the relationship between functional improvement and the EEG signal in the parietal area. Moreover, we did not find a prognostic EEG biomarker since none of the independent variable were significant for the analysis in which dependent variable was the baseline EEG power to predict the functional improvement.

## Discussion

### Clinical and Functional Outcomes

We found significant clinical improvement with the rehabilitation program, with the mean gain of 1.9 (SD:2.6) in WISCI and the mean gain of 4.1 (SD:5.7) in BBS. The training was well tolerated, and there were no adverse events.

### EEG Activity at Baseline Related to Functional Impairment at Baseline

High beta EEG activity in the central area had a negative correlation with gait and balance measured at baseline, in a way that greater high beta EEG power was related to worse clinical function. This relation was also significant in the frontal area, but not in parietal, temporal, or occipital areas. Beta oscillations have been associated with GABAergic activity in inhibitory interneurons and pyramidal neurons and are thought to reflect plastic changes in neural networks (Whittington et al., 2000; Rossiter et al., 2014). In a study comparing chronic SCI patients with age and sex matched, able-bodied control group, authors found consistently decreased alpha wave (8–13 Hz) and increased beta wave activity (13–30 Hz) in the SCI group (Herbert et al., 2007). Similarly, studies in stroke patients also showed a relation between increased beta wave activity and poor motor function (Simis et al., 2015; Thibaut et al., 2017). Our results are also supported by another study from our group, with a larger and different sample of incomplete SCI patients, in which we also found that greater high beta EEG power at baseline was negatively correlated with baseline gait function (Simis et al., 2020). Additionally, in that study, functional improvement was associated with greater baseline high beta power and greater decrease in high beta power after interventions. Even though we did not find any significant correlation between functional improvement and change in high beta power in the present study, this might be due to a type II error, considering our small sample size.

Overall, our results are in line with the literature and suggest that the correlation between baseline high beta EEG activity and poor functional impairment might be an indirect biomarker of adaptive/maladaptive changes and cortical reorganization within the corticomotor networks following SCI.

### Changes in EEG Activity (Post-Rehabilitation – Pre-Rehabilitation) Related to Functional Improvement

Change in alpha/theta activity ratio in the parietal area was negatively correlated with the improvement of gait (*p* = 0.049; adj-*R*^2^: 0.23) and balance (*p* = 0.043; adj-*R*^2^:0.24).

How alpha oscillations are generated remains unclear. Different models have been proposed including the cortical generation of the rhythms; TH as the pacemaker of the alpha rhythm (Bollimunta et al., 2011), or a more complex circuit (cortico-thalamo-cortical and intra-cortical circuits) associated with feedforward circuits that regulate alpha activity (Hindriks and van Putten, 2013). Previous studies comparing SCI participants with able-bodied controls showed that SCI participants have decreased alpha and increased beta activity in the cortex, with a larger difference in central, parietal and occipital areas. Besides, SCI subjects were consistently found to have lower peak frequencies than controls (Tran et al., 2004; Herbert et al., 2007). These EEG changes have been explained by the concept of thalamocortical dysrhythmia (TCD), which is the shift in the dominant spectral power to slower frequencies as a result of the deafferentation of excitatory input over TH (Llinas et al., 1999; Llinas and Steriade, 2006; Hirata and Castro-Alamancos, 2010). In our results, the negative correlation between functional improvement and alpha/theta ratio could be seen as contradictory, since it goes in the same direction of the TCD. On the other hand, it is important to note that in our results changes in alpha power was not correlated with functional improvement; but only its relation to theta power was, which is further discussed below. One explanation for why we did not see a direct correlation within the alpha band after rehabilitation might be explained by the disruption or the reorganization of the networks associated with motor learning, yet, in the absence of a control group this interpretation remains to be further explored.

The generation of theta oscillations has been related to structures in the hippocampus (hippocampal theta rhythm) and extra hippocampal (septal complex, entorhinal cortex, and pedunculopontine tegmentum). The functions of theta oscillation are not fully understood but are probably related to memory formation, sleep, and arousal (Hasselmo, 2005; Pignatelli et al., 2012). It is proposed that theta is promoted in the brainstem by GABAergic projection, from the neurons in *nucleus incertus* and *ventral tegmental nucleus of Gudden*, to the frontal cortex and nucleus accumbens. This circuit regulates the activity of thalamocortical neurons. It is suggested that GABAergic inputs to thalamic relay nuclei maintain tonic inhibition, which suppress unimportant information and unnecessary motor activity. On the other hand, when high attention is required, there is a decrease of this tonic inhibitory input allowing the enhanced arousal/attention and sensorimotor transmission, facilitating the motor response (Brown and McKenna, 2015). Therefore, the improvement of gait and balance may be related to the downregulation of this circuity (hence decrease in theta oscillations), which reflects as a decrease in alpha/theta activity in the parietal area. However, due to the absence of control group, it is not possible conclude whether these changes are solely related to motor recovery or RAGT, or both. It is possible that the EEG changes is associated to motor skill acquisition related to the therapy sessions, which may be present in subjects without SCI.

## Possible Clinical Applications

Our results suggest that EEG measures such as alpha/theta ratio might be a potential biomarker to determine treatment response and functional recovery in SCI patients and individualize rehabilitation therapies based neurophysiologic evidence. Additionally, these biomarkers can be potentially used as surrogate outcomes of patient’s clinical progression and to define discharge criteria from therapy. Moreover, it may be used to guide new rehabilitation therapies such as brain-computer-interface training and non-invasive brain stimulation techniques.

Even though we did not find any direct relationship between baseline EEG measures and functional outcomes, baseline EEG biomarkers have been implicated in the literature as a potential biomarker of treatment response in variety of neuropsychiatric diseases, such as multiple sclerosis (Tramonti et al., 2019) and depression (Olbrich and Arns, 2013) and therefore could play an important role in clinical practice. Further studies with larger sample sizes are needed to explore the potential use of baseline EEG measures as a predictor of treatment response in SCI.

## Limitations

The study had a small sample size with participants with different lesion levels and time since injury. Additionally, participants were taking medications that can influence EEG activity, which we were unable to control in our multivariate models due to our low sample size. Therefore, our results need to be confirmed in larger cohorts. Moreover, the results need to be carefully interpreted due to the absence of a control group. Another limitation is that EEG measures used in this study provides only an indirect measure of cortical activity and may not be sufficient to identify more local changes in the cortical structures. Future studies employing more advanced EEG analyses techniques to analyze the effects of rehabilitation techniques are needed to confirm our results. On the other hand, the use of simple and well-established methods (such as EEG power) can be more easily translated into clinical practice once proven to be accurate surrogate markers of brain activity in SCI.

## Conclusion

In SCI, functional impairment and subsequent improvement following rehabilitation therapy with RAGT correlated with the change in cortical activity measured by EEG. Our results suggest that EEG alpha/theta ratio may be a potential surrogate marker of functional improvement during rehabilitation. Future studies are necessary to improve and validate these findings as a neurophysiological biomarker for SCI rehabilitation.

## Data Availability Statement

The raw data supporting the conclusions of this article will be made available by the authors, without undue reservation.

## Ethics Statement

The studies involving human participants were reviewed and approved by Ethics Committee for Analysis of Research Projects (CAPPesq) of University of São Paulo Medical School. The patients/participants provided their written informed consent to participate in this study.

## Author Contributions

MS, TF, DR, LB, MI, and FF contributed conception and design of the study. MS, TF, DR, LB, and MI contributed for data the acquisition. MS and DD organized the database. MS and DD performed the statistical analysis. MS wrote the first draft of the manuscript. FF and DD wrote sections of the manuscript. All authors contributed to manuscript revision, read and approved the submitted version.

## Conflict of Interest

The authors declare that the research was conducted in the absence of any commercial or financial relationships that could be construed as a potential conflict of interest.
